# Chemokine Profile in Psoriasis Patients in Correlation with Disease Severity and Pruritus

**DOI:** 10.3390/ijms232113330

**Published:** 2022-11-01

**Authors:** Dorota Purzycka-Bohdan, Bogusław Nedoszytko, Monika Zabłotna, Jolanta Gleń, Aneta Szczerkowska-Dobosz, Roman J. Nowicki

**Affiliations:** 1 Department of Dermatology, Venereology and Allergology, Medical University of Gdansk, 80-214 Gdansk, Poland; 2 Invicta Fertility and Reproductive Centre, Molecular Laboratory, 81-740 Sopot, Poland

**Keywords:** chemokines, psoriasis, disease severity, pruritus

## Abstract

Psoriasis (PsO) is a chronic, immune-mediated, inflammatory skin disease associated in most cases with pruritus. Chemokines seem to play a significant role in PsO pathogenesis. The aim of the study was to analyse serum concentrations of CCL2/MCP-1, CCL3/MIP-1α, CCL4/MIP-1β, CCL5/RANTES, CCL17/TARC, CCL18/PARC, CCL22/MDC and CXCL8/IL-8, and their correlation with PsO severity and pruritus intensity. The study included 60 PsO patients and 40 healthy volunteers. Serum concentrations of six (CCL2/MCP-1, CCL3/MIP-1α, CCL5/RANTES, CCL17/TARC, CCL18/PARC and CCL22/MDC) out of eight analysed chemokines were significantly elevated in PsO patients; however, they did not correlate with disease severity. The serum level of CCL5/RANTES was significantly higher in patients with the psoriasis area and severity index (PASI) ≥ 15 (*p* = 0.01). The serum concentration of CCL17/TARC correlated positively with pruritus assessed using the visual analogue scale (VAS) (R = 0.47; *p* = 0.05). The study indicated CCL17/TARC as a potential biomarker of pruritus intensity in PsO patients. Chemokines appear to be involved in the development of PsO systemic inflammation. Further detailed studies on the interactions between chemokines, proinflammatory cytokines and immune system cells in PsO are required to search for new targeted therapies.

## 1. Introduction

Psoriasis (PsO) is a chronic, immune-mediated disease with a prevalence of approximately 2–3% in the general population. For many years, PsO was considered a skin condition with itchy, scaly patches, mostly located on the scalp, knees, elbows and torso. It has been shown that inflammatory process in the course of PsO may affect not only skin, but also various internal organs [1]. Therefore, PsO is currently regarded as a systemic inflammatory disorder with a long list of psychological, metabolic, arthritic, and cardiovascular comorbidities [2]. Moreover, approximetely 60–90% of PsO patients suffer from pruritus, which significantly reduces their quality of life [3,4]. Genetically programmed pathologic interaction between cells, cytokines and other biologic molecules triggered by environmental stimuli seems to be responsible for the complex PsO pathogenesis [5]. 

The disease is characterized by well-demarcated, scaly, erythematous lesions [6]. Activation of the immune system mediated by Th1 and Th17 lymphocytes, interleukin-12 (IL-12), IL-23, IL-17 and tumor necrosis factor-alpha (TNF-α) leads to the premature differentiation and hyperproliferation of keratinocytes, resulting in the development of scaling plaques [7]. Histologically, there is a pronounced infiltration of the skin by diverse types of cells, including CD4^+^ and CD8^+^ T lymphocytes, neutrophils, macrophages, mast cells and dendritic cells (DCs), which are a source of proinflammatory cytokines [8]. The synergistic effect of the cytokines likely explains most of the features of PsO, such as skin inflammation, hyperkeratinization and increased neovascularisation [6]. The implementation of new effective biological drugs in the treatment of PsO provides insights into how beneficial it is to block selected immune components, such as cytokines [9].

Chemokines, also known as chemotactic cytokines, are small protein molecules. Variation in their structure allows chemokines to be split into four subfamilies: CXC, CC, C and CX3C [10]. They are secreted in an inducible manner by various tissues and infiltrating leukocytes [11]. The secretion of chemokines is activated by the crucial proinflammatory cytokines, including IL-1, interferon *γ* (IFN-*γ*) and TNF-α [11,12]. Chemokines play an important role not only in the pathogenesis of many disorders, but also in a properly functioning organism [10]. In inflammatory processes, they act through their receptors and influence the migration of immune system cells. By participating in the activation of T lymphocytes, neutrophils and macrophages as well as their recruitment to the site of inflammation, chemokines seem to play a significant role in the pathogenesis of chronic inflammatory skin diseases, such as PsO [13,14]. Moreover, it is postulated that chemokines may also be involved in the aetiology of pruritus [15]. However, their impact on the development of PsO and the associated pruritus has not been fully investigated. The vast network of cells and cytokines that orchestrates the pathophysiology makes PsO complex to study. Understanding which cytokines play a pivotal role in PsO would direct further research and thus, suggest potential therapeutic targets [6].

The aim of the study was to evaluate the immune component in the pathogenesis of PsO by analysing the serum concentrations of selected chemokines (CCL2/MCP-1, CCL3/MIP-1α, CCL4/MIP-1β, CCL5/RANTES, CCL17/TARC, CCL18/PARC, CCL22/MDC, CXCL8/IL-8) and their correlation with the severity of the disease, as well as pruritus intensity in the PsO population of northern Poland.

## 2. Results

The PsO patients, when compared to the control group, showed a significantly higher serum level of CCL2/MCP-1, CCL3/MIP-1α, CCL5/RANTES, CCL17/TARC, CCL18/PARC and CCL22/MDC. However, no statistically significant differences were observed with respect to CCL4/MIP-1β and CXCL8/IL-8 (Table 1).

The analysis of the relationship between serum chemokine levels and disease severity as well as pruritus intensity, showed a positive correlation only in the case of CCL17/TARC for pruritus (Table 1). The study revealed a statistically significant correlation between the PASI score and VAS index (R = 0.34; *p* = 0.039).

When the PsO group was divided according to the onset of PsO (type I and II), disease severity (PASI) and family history of psoriasis, no differences were observed between the groups of patients defined in this way for most of the tested chemokines (data not shown). Differences were only noticed in the case of CCL5/RANTES.

**Figure 1:**
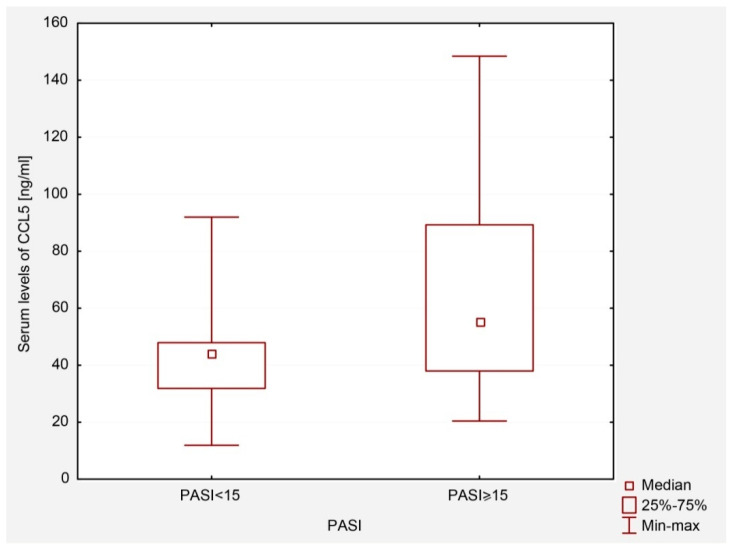
Serum levels of CCL5/RANTES in patients with PASI < 15 and PASI ≥ 15.

It was found that PsO patients with PASI ≥ 15 had a higher level of CCL5/RANTES than patients with less severe symptoms of the disease (PASI < 15), *p* = 0.01 (Figure 1).

## 3. Discussion

Our study supports the immune and inflammatory component in PsO. According to previous studies, the inflammatory process within PsO lesions results from the skin infiltration by leukocytes, mainly by T lymphocytes [16]. The recruitment, activation and migration of leukocytes to the site of inflammation is mediated largely by chemokines, which act as chemoattractants [17]. Just like in other chronic skin disorders, such as atopic dermatitis (AD), chemokines appear to be involved in the development of PsO inflammation [18]. The literature data showed an increased serum level of selected chemokines in PsO and indicated their potential role in the pathogenesis of the disease, which is in line with our results [11,19]. 

The monocyte chemoattractant protein-1 (MCP-1) as well as the macrophage inflammatory protein (MIP)-1α and MIP-1β are among the key chemokines regulating the migration and infiltration of monocytes/macrophages. Some studies reported an increased serum level of these chemokines in AD and PsO and highlighted their potential significance in the pathogenesis of both disorders [11,19]. As shown, serum CCL2/MCP-1 and CCL4/MIP-1β concentrations are significantly increased in AD patients compared to controls, and they are even higher in those with severe form of the disease [18]. Moreover, the authors noticed an augmented spontaneous production of CCL2/MCP-1, CCL3/MIP-1α and CCL4/MIP-1β by peripheral blood mononuclear cells (PBMC) in the course of AD [20]. The role of these three chemokines was also reported in PsO [21,22,23]. Dai et al. indicated the significance of CCL2/MCP-1, CCL3/MIP-1α and CCL4/MIP-1β in PsO aetiology as their serum levels were elevated in the studied group of 50 PsO patients vs. 50 controls and correlated positively with PASI [21]. Moreover, based on the study including 30 Caucasian patients and 10 controls, Lembo et al. confirmed an increased level of serum CCL2/MCP-1 in PsO [22]. The authors suggested this chemokine as a potential local inflammatory marker to assess the disease severity as well as anti-TNFα efficacy [22]. It was highlighted that anti-TNFα therapy reduced the expression of CCL2/MCP-1 within PsO skin robustly; however, it only moderately decreased CCL2/MCP-1 plasma concentration [22]. In our study, CCL2/MCP-1 and CCL3/MIP-1α in serum were also significantly elevated in PsO patients. Nevertheless, there were no significant differences in the serum level of CCL4/MIP-1β between patients and controls and no correlation with disease severity for these three analysed chemokines. 

The role of the regulated upon activation, normal T-cell expressed and secreted chemokine (CCL5/RANTES) in the development of eczematous and PsO lesions was highlighted in the literature. The studies demonstrated an increased serum CCL5/RANTES level in AD and PsO patients [20,24,25]. Moreover, Zabłotna et al. reported that selected -403 G/A *CCL5/RANTES* promoter gene polymorphisms may be risk factors for PsO and may influence its clinical presentation [24]. Zhao et al. presented a potential causal effect of elevated CCL5/RANTES concentration on the increased risk of PsO [25]. Based on the research that revealed a reduction of CCL5/RANTES expression in lesional PsO skin after narrow-band ultraviolet B (NB-UVB) therapy, Rateb et al. pointed to CCL5/RANTES as a potential marker of therapeutic efficacy [26]. We found that patients with PASI ≥ 15 had a higher serum concentration of CCL5/RANTES compared to those with less severe skin lesions. However, a randomized placebo-controlled clinical trial failed to show any significant clinical effect or any changes on immunohistochemical level in PsO patients treated with a placebo or a CCL5/RANTES receptor (CCR5) inhibitor [27]. This may indicate the complexity of the interactions between chemokines, other cytokines and immune cells rather than the role of one crucial factor in the development of PsO systemic inflammation.

Thymus and activation-regulated chemokine (CCL17/TARC) is a member of the T-helper 2 chemokine family. The pathogenic role of CCL17/TARC was suggested in skin diseases such as AD, cutaneous T-cell lymphoma, bullous pemphigoid, scabies and drug eruption [28,29,30]. The CCL17/TARC was indicated as a clinical biomarker in AD [31]. It was reported that CCL17/TARC may contribute to pruritus in AD patients, as its serum levels positively correlated with VAS [32]. Kawasaki et al. revealed that the serum CCL17/TARC level can potentially be one of the biomarkers reflecting the severity of systemic inflammation in PsO patients, although not as much as in patients with AD [33]. We did not observe a significant reletionship between the systemic CCL17/TARC level and PASI. However, we found that the serum concentration of CCL17/TARC in PsO patients correlated positively with the intensity of pruritus measured with VAS, and the *p*-value (*p* = 0.05) was on the borderline of statistical significance. This may indicate the role of CCL17/TARC as a potential biomarker of pruritus intensity not only in AD, but also in PsO patients. However, further studies on a larger group of PsO patients are necessary. 

The pulmonary and activation-regulated chemokine (CCL18/PARC) is produced by epidermal and dermal antigen-presenting cells as well as by keratinocytes. Skin-homing human T cells express the CCR8 receptor. It has been found that CCL18/PARC binds with the CCR8 receptor and induces homing of T cells in inflammatory skin lesions [34]. Increased levels of CCL18/PARC have been linked to allergic contact hypersensitivity, AD and other chronic inflammatory conditions [34]. According to the article of Kim et al., CCL18/PARC mRNA was significantly increased in acute AD lesions and in PsO plaques; however, it was lower in the nonaffected skin of patients with AD [35]. Moreover, the study revealed that the serum levels of CCL18/PARC were not different in patients with AD and PsO. We also noticed a significantly increased serum concentration of this chemokine in PsO patients. However, the level of CCL18/PARC did not correlate with the severity of the disease and pruritus. This may suggest a supporting role of CCL18/PARC in the inflammatory process in PsO.

Macrophage-derived chemokine (MDC), newly termed CCL22, is a CC chemokine that potentially serves as a chemoattractant for monocytes, monocyte-derived dendritic cells (DCs) and natural killer (NK) cells [36]. The CCL22/MDC was reported to be involved in the pathogenesis of AD. The study performed by Kakinuma et al., including 45 patients with AD, 25 patients with PsO and 25 healthy controls, revealed that serum levels of CCL22/MDC in AD patients were significantly higher than those in healthy controls and PsO patients [36]. Moreover, the authors noticed the correlation between CCL22/MDC and AD activity. Not much is known about the role of CCL22/MDC in the pathogenesis of PsO. Kusumoto et al. observed that increased CCL22/MDC expression in PsO skin predicts a good response to infliximab therapy [37]. In our study, we noticed a significantly higher serum concentration of CCL22/MDC in PsO patients compared to controls; however, it was not statistically significant in correlation with PsO severity.

The IL-8, also known as CXCL8, is a chemokine produced by macrophages and other cells, such as endothelial and epithelial cells [11,38,39]. The CXCL8/IL-8 secretion appears to be stimulated by proinflammatory cytokines, such as TNF-α and IL-17 [38]. It acts as a chemotactic cytokine of neutrophils, causing their migration to the skin and the formation of microabsceses in the course of PsO. This cytokine has the ability to bind with two receptors, CXCR1 and CXCR2, which are not selective for CXCL8/IL-8 [40]. These receptors are also capable of binding with CX3CL1 and CXCL5, respectively; thus, bypassing the pathway of their activation by CXCL8/IL-8 [11,38]. Therefore, blocking the CXCL8/IL-8 itself could prove ineffective. The literature data indicate a role of CXCL8/IL-8 at local level in PsO skin. Lemster et al. reported CXCL8/IL-8 expression in the affected skin of all the PsO patients included in the study, but not in the clinically normal skin of healthy subjects [41]. Moreover, CXCL8/IL-8 mRNA was not detected in the skin of any patient after the commencement of systemic tacrolimus therapy. The authors suggested the CXCL8/IL-8 pathway may be an important mechanism underlying the therapeutic efficacy of tacrolimus [41]. The impact of NB-UVB therapy on CXCL8/IL-8 was also reported. Although no statistically significant differences were detected in CXCL8/IL-8 serum levels between the controls and PsO patients before treatment, after NB-UVB therapy, the serum concentrations of this chemokine in PsO patients were significantly decreased compared to the controls [42]. Sticherling et al. suggested CXCL8/IL-8 had a local rather than systemic function, explaining that in PsO either the serum CXCL8/IL-8 was absent or some mechanisms were effectively binding and/or inactivating CXCL8/IL8 as it entered circulation [43]. Similarly, in our study, in terms of the serum concentration of the chemokine, no difference was observed between the PsO group and the control group.

Most chemokines involved in the PsO development are secreted by keratinocytes. Activated keratinocytes are also a source of antimicrobial peptides and proinflammatory cytokines, which together with chemokines contribute to the intensification of inflammation [44]. Recent studies highlighted the significance of newly established protein markers of PsO. The multifunctional role of the S100 protein family was suggested in PsO pathogenesis [44,45,46]. S100 proteins have been shown to act as alarmins (DAMPs), antimicrobial peptides, proinflammation stimulators as well as chemoattractants [45]. S100 members can induce neutrophil chemotaxis and stimulate neutrophils to release cytokines (TNF-α and IL-6) and chemokines (CCL2, CCL3, CCL4 and CXCL8), leading to the intensification of the proinflammatory signalling cascade [45,47]. Some authors suggested S100 proteins as potential biomarkers for PsO severity and novel therapeutic targets [44,46]. Moreover, other studies revealed the role of further proteins in the aetiology of PsO. Based on clinical and immunohistochemical research, El Dein Mohamed at al. identified the valosine-containing protein (VCP) as a promising marker for the follow-up and monitoring of PsO [48]. The VCP, by activating the nuclear factor κB (NF-κB) and inflammatory cytokines, contributes to the imbalance of cytokines and promotes the inflammatory process in organs. Recent data shed new light on the relationship between the systemic inflammation of PsO and cardio-metabolic syndrome, providing an insight into novel key players: proteins such as proprotein convertase subtilisin/kexin type-9 (PSCK9), angiopoietin-like protein 8 (ANGPLT8), sortilin (SORT1) and cholesteryl ester transfer proteins (CEPT) [49]. These reports point to the complexity of the immunological pathways in PsO and indicate the need for further studies on the interactions between chemokines, cytokines and other proteins in the disease pathogenesis.

Although our research focused on the relationship of serum-selected chemokines level and PsO severity as well as an itch intensity, we additionally performed a correlation analysis between the PASI score and VAS index. In our group of patients, a statistically significant positive correlation between PsO severity and pruritus intensity was observed. The literature data on the interplay between these two factors remain inconclusive. According to Szepietowski et al., there is a significant correlation between the PASI score and the intensity of itch assessed by both scales: VAS and the questionnaire method (r = 0.29, *p* < 0.01 for both analyses) [50]. Studies performed by Damiani et al. [51] and Bahali et al. [52] also supported this relationship. In another research, Jaworecka et al. observed that the pruritus intensity increased along with PsO severity; however, the statistical significance was confirmed only in palmoplantar pustular PsO and scalp PsO (*p* < 0.05) [3]. Other authors, however, noted no significant correlation between PsO severity and pruritus [53,54,55].

Some limitations of our study should be considered. Firstly, the study did not include all known chemokines. Secondly, the results need further evaluation in larger prospective studies. Nevertheless, by assessing the chemokine profile in a group of PsO patients, we demonstrated the participation of several of them in the development of the disease. The lack of positive correlation between chemokine serum levels and disease severity may suggest a supportive rather than a key role of the analysed chemokines in systemic inflammation in the course of PsO; however, further studies on a larger group of patients are required to confirm these observations.

## 4. Materials and Methods

### 4.1. PsO Group

The PsO group included 60 adults (30 men and 30 women; mean age: 48.5 years, range: 18–76 years), unrelated patients with chronic plaque PsO who were admitted to the Department of Dermatology as well as to the Dermatology Outpatient Clinic of the Medical University of Gdansk. The recruited patients had not been treated systemically for PsO (retinoids, cyclosporine, methotrexate, photochemotherapy) at least for the previous 3 months and had not received topical medications for the previous 1 week. Patients suffering from other chronic dermatoses, systemic inflammatory disorders or malignancies as well as those treated with biological drugs were excluded.

### 4.2. Control Group

The control group included 40 adults (29 men and 11 women; mean age: 39.75 years, range: 18–84 years), healthy, unrelated volunteers, without PsO or other systemic chronic inflammatory skin diseases. The exclusion criterion from the control group was also a positive family history of PsO.

All the participants were exclusively of Polish descent (the population of northern Poland). The characteristics of the participants are presented in Table 2. 

### 4.3. Classification into Early and Late-Onset PsO

Patients were divided into two subgroups: early-onset PsO (the PsO onset age < 40 years; *n* = 45) and late-onset PsO (the PsO onset age ≥ 40 years; *n* = 15) [56].

### 4.4. Assessment of PsO Severity

The PsO diagnosis was based on detailed dermatological examination. The disease severity assessment was performed using the psoriasis area and severity index (PASI, range 0–72).

### 4.5. Assessment of Pruritus

The intensity of pruritus was measured with the visual analogue scale (VAS) as an average itch from the previous week (scale from 0 to 10) in all the participants [57]. 

### 4.6. Evaluation of Serum Chemokine Levels

Serum levels of CCL2/MCP-1, CCL3/MIP-1α, CCL4/MIP-1β, CCL5/RANTES, CCL17/TARC, CCL18/PARC, CCL22/MDC and CXCL8/IL-8 were analysed in all the PsO participants and controls. The serum levels were assessed using an enzyme-linked immunoabsorbent assay (ELISA) standard kit (BioVendor-Laboratorni medicina a.s., Brno, Czech Republic; R&D Systems, Inc. Minneapolis, MN, USA; Diaclone SAS, Besancon, France). These products had been tested by quality control and passed internal specifications. All the procedures followed the manufacturer’s instructions. 

### 4.7. Statistical Analysis

Statistical calculations were performed using the Statistica 12.0 software package (StatSoft, Inc., Tulsa, OK, USA, 2015). The analysis of qualitative features was performed using the χ^2^ test applying the Pearson method. The normality of the data distribution was tested with the W Shapiro-Wilk test. Normal distribution variables were analysed using the Student’s *t*-test. Variables that did not meet the assumptions of the parametric tests were analysed using non-parametric tests (ANOVA equivalents): the Mann–Whitney U test (comparisons of two trials) or the Kruskal-Wallis test (comparisons of multiple trials). The correlation coefficients were evaluated using the Spearman’s rank correlation test. A *p*-value < 0.05 was considered to be statistically significant. 

## 5. Conclusions

In summary, our results support the concept of a systemic and an inflammatory component in PsO. Chemokines appear to be involved in the complex aetiology of the disease. Moreover, the presented study puts emphasis on the possible role of CCL17/TARC as a potential biomarker of pruritus intensity in PsO patients. Nevertheless, further detailed studies on the interactions between the analysed chemokines, proinflammatory cytokines and immune system cells are needed to search for new effective targeted therapies for PsO.

## Figures and Tables

**Table 1:** Serum concentrations of chemokines in correlation with disease severity and pruritus intensity in PsO.

Chemokines	Controls *n* = 40 Mean ± SD Median [Range] (pg/mL)	PsO *n* = 60 Mean ± SD Median [Range] (pg/mL)	PsO vs. Controls *p*-Value	PsO Correlation between Chemokine Serum Level and PASI R * p-Value	PsO Correlation between Chemokine Serum Level and Pruritus Assessed by VAS R * p-Value
CCL2 (MCP-1)	301.21 ± 88.40 303.00 [130.4–544.4]	415.71 ± 138.06 409.95 [205.4–934.5]	<0.0001	NS	NS
CCL3 (MIP-1α)	13.31 ± 7.98 10.60 [2.9–32.2]	31.15 ± 23.19 24.47 [6.1–136.8]	<0.0001	NS	NS
CCL4 (MIP-1β)	74.47 ± 31.72 73.75 [13.9–148.3]	95.29 ± 70.55 75.10 [21.3–426.3]	0.32	NS	NS
CCL5 (RANTES)	44,471 ± 24,786.8 42,804.00 [1877–94,522]	68,695.78 ± 91,785 47,144.00 [11,914–148,425]	0.037	NS	NS
CCL17 (TARC)	401.48 ± 182.40 408.59 [66.1–950.6]	665.96 ± 463.24 510.20 [70.9–2300]	0.002	NS	R = 0.47 *p* = 0.05
CCL18 (PARC)	12.80 ± 6.72 11.85 [3.3–39.8]	49.76 ± 26.56 42.48 [8.2–125]	<0.0001	NS	NS
CCL22 (MDC)	835.55 ± 336.96 770.46 [238.1–2262.9]	1463.62 ± 816.39 1279.26 [254.4–3770.7]	<0.0001	NS	NS
CXCL8 (IL-8)	12.36 ± 7.41 9.44 [2.6–38.1]	13.85 ± 14.80 9.62 [2.3–76]	0.62	NS	NS

* PsO—Psoriasis, SD—Standard Deviation, PASI—Psoriasis Area and Severity Index, VAS—Visual Analogue Scale, R—Spearman’s rank correlation coefficient, NS—Not Significant.

**Table 2:** Characteristics of the PsO and control group.

Parameter	PsO	Early-Onset PsO	Late-Onset PsO	Controls
Total number of subjects, n (%)	60	45 (75.0)	15 (25.0)	40
Males, n (%)	30 (50.0)	26 (57.8)	4 (26.7)	29 (72.5)
Females, n (%)	30 (50.0)	19 (42.2)	11 (73.3)	11 (27.5)
Age at enrolment (years) Mean ± SD	48.5 ± 14.6	45.3 ± 14.9	58.1 ± 7.8	39.75 ± 15.03
Positive family history of PsO, n (%)	29 (48.3)	22 (75.9)	7 (24.1)	0
PASI (points) Mean ± SD	16.3 ± 9.2	16.9 ± 9.3	14.6 ± 9.0	-
Pruritus according to VAS Mean ± SD	5.5 ± 1.8	5.4 ± 1.9	6.0 ± 1.7	0

PsO—Psoriasis, SD—Standard Deviation, PASI—Psoriasis Area and Severity Index, VAS—Visual Analogue Scale.

## Data Availability

The data that support the findings of this study are available from the corresponding author upon reasonable request.
